# Oral Phosphatidylcholine Improves Intestinal Barrier Function in Drug-Induced Liver Injury in Rats

**DOI:** 10.1155/2019/8723460

**Published:** 2019-09-02

**Authors:** Meijuan Chen, Haijun Huang, Pengcheng Zhou, Jiajie Zhang, Yining Dai, Danhong Yang, Xuegong Fan, Hongying Pan

**Affiliations:** ^1^Department of Infectious Disease, Zhejiang Provincial People's Hospital, People's Hospital of Hangzhou Medical College, Hangzhou, Zhejiang 310014, China; ^2^Infection Control Center, Xiangya Hospital, Central South University, Changsha, Hunan 410008, China

## Abstract

**Objective:**

Phosphatidylcholine (PC) is the major surface-active phospholipid and creates a hydrophobic nature to the surface. It has been reported to reverse the progression of liver fibrosis and to improve liver function. The aim of the present study was to evaluate the effects of orally administered PC on intestinal barrier function (IBF) in rats with drug-induced liver injury.

**Method:**

Rats with carbon tetrachloride- (CCl4-) induced liver injury were treated with 100 mg/kg PC once daily for 21 days. The effects of PC therapy on (i) liver function and portal pressure, (ii) intestinal and hepatic histology, and (iii) plasma endotoxin, diamine oxidase (DAO), and tumour necrosis factor- (TNF-) *α* levels were investigated.

**Results:**

PC therapy reduced portal pressure and improved the liver function in CCl4-induced liver injury. In PC-treated liver injury rats, collagen fibres were gradually decreased, while the disordered arrangement of hepatocytes and disorganized hepatic lobules were partially repaired, and inflammatory cell infiltration was decreased in the fibrous tissue. Lower inflammatory cell infiltration in the ileum improved intestinal histology, and reduced serum DAO levels were observed in PC-treated cirrhotic rats. These changes were associated with reduced inflammatory activity, as indicated by decreased serum TNF-*α* levels and plasma endotoxin levels.

**Conclusions:**

These results suggest that PC therapy is hepatoprotective and is able to restore IBF and reduce endotoxaemia in rats with drug-induced liver injury.

## 1. Introduction

Liver disease is a substantial health burden worldwide and is caused by a variety of factors. Drug-induced liver injury represents a wide range of responses following exposure to any manufactured or naturally occurring chemical compound. Previous experimental and clinical studies suggested that liver injury, especially cirrhosis, is associated with increased gut permeability and intestinal barrier dysfunction [1–3]. Increased permeability of the intestinal barrier may result in bacterial translocation and endotoxaemia [4, 5], which could induce liver dysfunction and even contribute to systemic haemodynamic derangement, resulting in the diverse complications of cirrhosis [5]. Therefore, repairing the intestinal barrier is of great importance in the treatment of liver injury.

Phosphatidylcholine (PC) is a ubiquitous membrane phospholipid that is essential for cellular differentiation, proliferation, and regeneration and is necessary for the transport of molecules through membranes [6]. It has been recognized as a marker of liver diseases [7]. Levels of PC in both serum and liver tissue are markedly decreased in liver cirrhosis [8, 9]. According to previous studies, PC treatment in experimental models of liver injury has been determined to reverse the progression of liver fibrosis [10] and to improve liver function [11].

Surface-active PCs are important for the hydrophobic biophysical surface properties of the intestine, providing a barrier against microorganisms and toxic by-products [12]. Previous studies have demonstrated that exogenous PCs prevent mucosal damage induced by nonsteroidal anti-inflammatory drugs or lipopolysaccharides in the stomach, duodenum, and small intestine [12, 13]. Previous research performed by the authors of the present study demonstrated that faecal lysophosphatidylcholine content was markedly increased in patients with liver cirrhosis or hepatocellular carcinoma [14, 15], thus indicating a decrease in PC in the intestinal lumina. However, the effect of PC on the intestinal barrier in drug-induced liver injury remains unknown. The aim of the present study was to evaluate the effect of orally administered PC on the intestinal barrier functions of rats with carbon tetrachloride- (CCl4-) induced liver injury.

## 2. Material and Methods

### 2.1. Animals

A total of 40 healthy male Sprague-Dawley rats weighing 180-220 g were provided by the Department of Animal Care, Zhejiang Traditional Medicine University (Hangzhou, China). Rats were fed with standard laboratory food and water ad libitum. The temperature in the feeding room was kept between 20°C and 25°C, and the relative humidity was 50%-65%. All experimental protocols were approved by the Institutional Animal Care and Use Committee of Zhejiang Provincial People's Hospital (Hangzhou, China).

### 2.2. Experimental Protocol

Rats were randomly divided into the normal control (healthy rats (HR); *n* = 10) and liver injury groups. Liver injury was induced in 30 rats via subcutaneous injection of 50% CCl4 (Kelong Chemical Reagent Company, Chengdu City, Sichuan, China) in a 1 : 1 mixture with olive oil (1 ml/kg) twice a week for eight weeks. The animals with liver injury were subsequently randomized into the treatment (LI-PC; *n* = 15) or the vehicle (LI; *n* = 15) group. The LI-PC group was administered intragastrically with 100 mg/kg PC (Sigma-Aldrich; Merck kGaA, Darmstadt, Germany; Catalogue No. 1535755) once daily for 21 days. The HR group received the vehicle (1 ml saline) and was analysed in parallel.

Following the treatment period, rats underwent laparotomy. They were anaesthetized with 50 mg/kg pentobarbital sodium subcutaneously, and a midline incision was made to measure portal pressure. Subsequently, blood and tissue samples from the left major liver lobe and the ileum were collected. All rats were subsequently sacrificed by intraperitoneal injection of 8 ml/kg body weight of 10% chloral hydrate (Aoxin Reagent Company, Yangzhou City, Jiangsu, China). Tissue samples were washed with phosphate-buffered saline, harvested, and fixed at room temperature overnight in 10% formalin for histological examination. Tissue specimens were frozen immediately following fixation via immersion in liquid nitrogen and stored at -80°C. Blood samples were evaluated to determine biochemical parameters.

### 2.3. Assessment of Histopathological Changes

Histopathological assessments were made by pathological experts. Changes in liver histopathology were assessed by haematoxylin and eosin staining, whereas Masson's trichrome stain was used to assess the degree of fibrosis.

Ileal biopsies were processed, and the sections were stained with haematoxylin and eosin prior to examination with a light microscope. Ultrastructural evaluation of the ileal biopsies was performed following a standard procedure, wherein biopsies were immediately fixed at room temperature overnight with 2.5% glutaraldehyde, postfixed with 1% osmium tetroxide, and embedded in resin (EMbed 812 Embedding kit; Electron Microscopy Sciences, Hatfield, PA, USA). Ultrathin sections were cut at a thickness of 120 nm and subsequently stained with lead citrate and uranyl acetate. A transmission electron microscope (Philips Tecnai 10; FEI, Thermo Fisher Scientific Inc., Waltham, MA, USA) and an electron microscope image analyser (Erlangshen ES500W; Gatan Inc., Pleasanton, CA, USA) were used to analyse the samples.

### 2.4. Liver Function

To separate the serum for liver function analysis, blood samples were centrifuged at 3,000 × g for 10 min at room temperature. Serum samples were analysed using a Hitachi 7600 automatic analyser (Hitachi Ltd., Tokyo, Japan).

### 2.5. Portal Pressure Determination

During laparotomy, portal pressure was monitored continuously and recorded using a strain-gauge transducer connected to the portal inflow cannula, 6 cm proximal to the perfusion cannula, using a BL-420S data acquisition and analysis system (Chengdu TME Technology Co. Ltd., Chengdu, China), following a previously published method [16]. The results are presented as mmHg.

### 2.6. Measurement of Endotoxin, TNF-*α*, and Diamine Oxidase

A quantitative chromogenic limulus amebocyte lysate assay (Aihua Medical, Shanghai, China) was performed to determine plasma endotoxin, according to the manufacturer's protocol [17]. The detection limit of endotoxin was 0.2 units, and the results are presented as EU/ml. Tumour necrosis factor- (TNF-) *α* levels in rat serum were detected using an ELISA kit (RAB0480, Sigma-Aldrich; Merck kGaA) and presented as ng/ml. The concentration of serum diamine oxidase (DAO), a biomarker of intestinal barrier dysfunction, was determined using ultraviolet spectrophotometry (UV-2102C, Juchne, USA).

### 2.7. Statistical Analysis

The results are presented as the mean ± standard deviation. Statistical analyses were performed using one-way analysis of variance. Post hoc comparisons were performed with the Duncan or Mann-Whitney nonparametric tests. Data were analysed using SPSS for Windows (ver. 16.0; SPSS Inc., Chicago, IL, USA). *P* ≤ 0.05 was considered to indicate a statistically significant difference.

## 3. Results

### 3.1. Liver Function and Portal Pressure

Liver function indices and portal pressure in the three groups are listed in Table 1. There were significant increases in alanine aminotransferase, aspartate aminotransferase, and total bilirubin levels (*P* < 0.05) and decreases in total protein levels (*P* < 0.05) in the untreated LI group compared with the LI-PC and HR control groups. Alanine transaminase and aspartate aminotransferase levels were higher in the LI-PC group than in the HR group, although these differences were not significant. Portal pressure was significantly higher in the LI group than in the HR group (*P* < 0.05), whereas a significantly lower portal pressure was exhibited by the LI-PC group than by the LI group (*P* < 0.05).

### 3.2. Liver Histopathology

**Figure 1:**
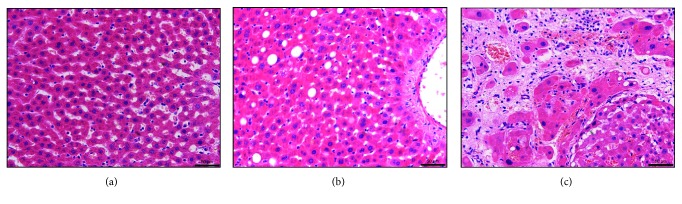
Light micrograph of hepatic histopathology stained with haematoxylin and eosin (magnification, ×200): (a) normal control group; (b) liver injury and treated with phosphatidylcholine group; (c) liver injury group.

As presented in Figure 1, hepatocytes exhibited a disordered arrangement in the LI group, with extensive fatty degeneration, marked swelling, spotty necrosis, and eosinophilic bodies apparent in the hepatic tissue. Collagen fibres divided the disorganized lobules, and abundant lymphocytes infiltrated the fibrous tissue with proliferation of bile ducts. In LI-PC rats, collagen fibres were gradually decreased, damaged hepatic lobules were partially repaired, fatty degeneration of hepatocytes was reduced, and inflammatory cell infiltration was decreased in the fibrous tissue.

### 3.3. Ileal Histopathology

**Figure 2:**
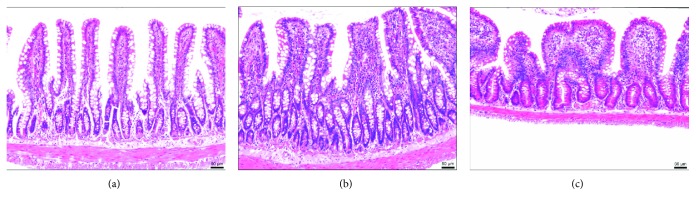
Light micrograph of ileal histopathology stained with haematoxylin and eosin (magnification, ×100): (a) normal control group; (b) liver injury and treated with phosphatidylcholine group; (c) liver injury group.

Light microscopic analysis of Paneth cells detected a decrease in the LI-PC group and a greater decrease in the LI group, compared with the HR group. Submucosal oedema was not obvious in LI-PC rats, while it was noticeable in the LI group. Inflammatory cell infiltration was lower in the LI-PC group than in the LI group (Figure 2).

**Figure 3:**
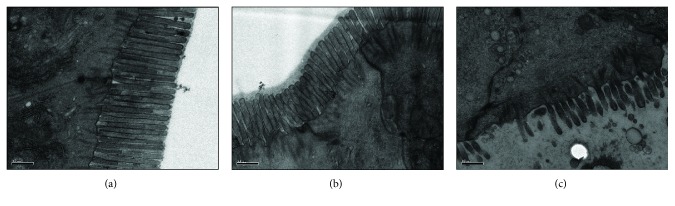
Transmission electron microscopy of ileal histopathology (magnification, ×15,000): (a) normal control group; (b) liver injury and treated with phosphatidylcholine group; (c) liver injury group.

Transmission electron microscopy revealed widened intercellular junctions, distorted microvilli, and increased and dilated mitochondria in LI rats. In LI-PC rats, the microvilli and mitochondria in the intestinal epithelium were normal and the intercellular junctions thicker (Figure 3).

### 3.4. Plasma Endotoxin and Serum DAO and TNF-*α* Levels

Plasma endotoxin and serum DAO and TNF-*α* levels are presented in Table 2. Serum DAO was significantly higher in LI rats than in HR rats (*P* < 0.05) and LI-PC rats (*P* < 0.05). The reduced serum DAO concentration in LI-PC rats was associated with lower serum inflammatory activity, as demonstrated by the significantly decreased serum TNF-*α* and plasma endotoxin levels in the LI-PC group compared with the LI group (*P* < 0.05). However, serum TNF-*α* and plasma endotoxin levels were significantly higher in the LI-PC group than in the HR group (*P* < 0.05).

## 4. Discussion

Cell mucous membrane is overlaid with a coating of phospholipid, which creates a hydrophobic nature to the surface. PC is the major surface-active agent. Therefore, maintenance of PC, which is primarily constituting the hydrophobic surface, can be of great importance for physical health and prevention of diseases.

It has been demonstrated that PC is hepatoprotective in rats with experimentally induced liver injury [11]. The results of the present study are in accordance with the previous literature, as improved liver function was demonstrated in rats with liver injury receiving PC treatment. Furthermore, PC-treated rats exhibited significantly lower portal pressure than untreated rats with liver injury. Additionally, liver histopathological changes were improved in LI-PC rats as collagen fibres gradually decreased and damaged hepatic lobules were partly repaired by PC treatment. These results indicated that PC treatment reduced liver fibrosis [10, 11] and the decrease in portal pressure in LI-PC rats was due to attenuated fibrogenesis and hepatoprotective effects.

The present study demonstrated that intestinal histopathological changes were improved following administration of PC to rats with drug-induced liver injury. Light microscopy revealed that the decreases in Paneth cells, submucosal oedema, and inflammatory cell infiltration were partially prevented by PC treatment. Transmission electron microscopy indicated that microvilli and mitochondria appeared normal and that the intercellular junctions were widened in LI-PC rats. Serum DAO activity and endotoxin levels are useful markers of intestinal mucosal permeability, intestinal injury, and reperfusion insults [18]. In parallel with the intestinal histopathological findings, it was observed that serum DAO activity and plasma endotoxin concentrations were significantly lower in rats treated with PC than in untreated rats. The favourable effects of PC on the intestine may be partially explained by the reduction in portal pressure observed in LI-PC rats, as well as direct protection of mucosal barrier integrity.

It has been demonstrated that rats with liver cirrhosis have markedly higher serum TNF-*α* levels [19], which is in agreement with the present results. The altered intestinal barrier function (IBF) in rats with liver injury was associated with upregulation of serum TNF-*α* levels. Increased TNF-*α* expression in the guts of cirrhotic rats may be the result of increased intestinal permeability, permitting the penetration of bacteria and endotoxins into the gut wall. Therefore, the reduction in serum TNF-*α* levels observed in LI-PC rats may reflect improvements in IBF. However, previous publications have suggested that exogenous PC targets inflammatory reactions in the gastrointestinal tract [20, 21]. These findings [20, 21] suggest that PC treatment may exert direct anti-inflammatory activity in the gut, contributing to the protection of IBF.

The benefit of PC on IBF in cirrhotic rats was due to a reduction in portal pressure, potentially resulting from influences on intestinal barrier structure and function. PC is integral to tissue membranes, cells, and organelles in the intestinal mucosa, as has been supported by the observation that PC prevents enteric bacterial translocation during the early stage of experimental acute liver failure in rats [22]. Furthermore, endogenous PC is necessary for mucosal hydrophobicity and contributes to the barrier properties of the epithelium [12, 13]. It has been determined that PC acts by direct adsorption in the mucus layer, thereby enhancing barrier properties [13]. Therefore, the improvement in IBF observed in drug-induced liver injury treated with PC may depend on direct trophic effects in the gut wall and maintenance of the intestinal barrier.

The present findings suggest that PC therapy is able to restore IBF and reduce endotoxaemia in drug-induced liver injury in rats. Future clinical trials are required to determine whether this lipid is able to profitably influence the natural history of drug-induced liver injury and reduce the risk of bacterial translocation and other bacterial complications in patients with liver injury or cirrhosis.

## Figures and Tables

**Table 1:** Comparison of liver function and the value of portal pressure among the experimental groups.

Parameters	HR	LI-PC	LI
ALT (U/l)	55.12 ± 15.55^b^	76.40 ± 29.14^b^	245.36 ± 160.39^a^
AST (U/l)	141.01 ± 42.10^b^	147.60 ± 35.65^b^	497.51 ± 360.02^a^
TB (*μ*mol/l)	0.21 ± 0.13^b^	0.21 ± 0.11^b^	3.03 ± 3.64^a^
TP (g/l)	63.50 ± 4.04^b^	61.97 ± 5.03^b^	58.47 ± 4.13^a^
Portal pressure (mmHg)	5.54 ± 1.87^b^	10.24 ± 1.04^a,b^	12.77 ± 0.70^a^

Data are expressed as the mean ± standard deviation. ^a^*P* < 0.05 vs. the HR group; ^b^*P* < 0.05 vs. the LI group. HR: normal control group; LI: liver injury group; LI-PC: liver injury and treated with phosphatidylcholine group; ALT: alanine transaminase; AST: aspartate aminotransferase; TB: total bilirubin; TP: total protein.

**Table 2:** Comparison of the plasma endotoxin level and serum DAO activities and TNF-*α* levels among the experimental groups.

Parameters	HR	LI	LI-PC
Endotoxin (EU/ml)	0.37 ± 0.06^b^	0.90 ± 0.07^a^	0.52 ± 0.06^a,b^
DAO (U/l)	4.96 ± 1.95^b^	6.96 ± 2.09^a^	5.45 ± 2.06^b^
TNF-*α* (pg/ml)	6.38 ± 4.30^b^	50.08 ± 28.55^a^	11.82 ± 4.34^a,b^

Data are expressed as the mean ± standard deviation. ^a^*P* < 0.05 vs. the HR group; ^b^*P* < 0.05 vs. the LI group. HR: normal control group; LI: liver injury group; LI-PC: liver injury and treated with phosphatidylcholine group; DAO: diamine oxidase; TNF: tumour necrosis factor.

## Data Availability

In the manuscript titled “Oral Phosphatidylcholine Improves Intestinal Barrier Function in Drug-Induced Liver Injury in Rats,” all data generated or analysed during this study are included in this article. You can access the data in Results, tables, and figures.
